# Association between fibrinogen-to-albumin ratio and the presence and severity of coronary artery disease in patients with acute coronary syndrome

**DOI:** 10.1186/s12872-021-02400-z

**Published:** 2021-12-07

**Authors:** Zhenzhen Duan, Chaodi Luo, Bowen Fu, Dan Han

**Affiliations:** 1 Department of Peripheral Vascular Diseases, the First Affiliated Hospital of Xi’an Jiaotong University, Xi’an, People’s Republic of China; 2 Department of Cardiology, the First Affiliated Hospital of Xi’an Jiaotong University, Xi’an, People’s Republic of China; 3 Department of Cardiovascular Surgery, the First Affiliated Hospital of Xi’an Jiaotong University, 277 Yanta West Road, Xi’an, 710061 Shaanxi People’s Republic of China

**Keywords:** Fibrinogen to albumin ratio, Gensini score, Acute coronary syndrome

## Abstract

**Objective:**

Although the levels of plasma fibrinogen and albumin have been proven to be in relation to coronary heart disease (CHD), the association between fibrinogen-to-albumin ratio (FAR) and acute coronary syndrome (ACS) has not been adequately investigated. The aim of this study is to investigate the relationship between FAR and the presence and severity of CHD in patients with ACS.

**Methods and results:**

A total of 1575 individuals who received coronary angiography (CAG) were enrolled. Patients were divided into the ACS group and the control group. The severity of ACS was determined by Gensini score, number of diseased coronary artery and the presence of myocardial infarction (MI). Data showed that the level of FAR in ACS group was higher than in the control group (81.20 ± 35.45 vs. 72.89 ± 20.24, *P* < 0.001). The results from subgroup analysis indicated that the values of FAR in the high Gensini score group, MI group and multiple-vessel stenosis group were higher than the matched subgroups. After adjustment for confounders, FAR was still independently related to the presence and severity of ACS (MI OR 2.097, 95%CI 1.430–3.076; High GS: OR 2.335, 95%CI 1.567–3.479; multiple-vessel disease: OR 2.088, 95%CI 1.439–3.030; *P* < 0.05).

**Conclusion:**

The levels of FAR are independently associated with the presence and the severity of coronary artery disease in patients with ACS. Furthermore, FAR, as a more convenient and rapid biological indicator, may provide a new idea for predicting the presence and severity of ACS.

**Supplementary Information:**

The online version contains supplementary material available at 10.1186/s12872-021-02400-z.

## Introduction

Coronary heart disease (CHD) is a kind of ischemic cardiovascular condition caused by coronary atherosclerosis which affects vessel wall at different degrees. Although the prevalence of CHD has decreased over the past decade, it remains the leading cause of mortality worldwide [1]. Acute coronary syndrome (ACS), a common and a more severe type of CHD, is a consequence of acute coronary thrombotic occlusion followed by myocardial ischaemia and necrosis [2, 3]. It is acknowledged that there is a strong relationship between ACS and cardiovascular risk factors such as hypertension, diabetes, hyperlipidemia and inflammation [4–8]. Recent studies proposed that the ratio of fibrinogen-to-albumin (FAR) provided a simple and feasible laboratory method for predicting the severity of atherosclerosis, however, the association between FAR and the extent and severity of ACS has not been thoroughly studied.

Fibrinogen is an indicator of the state of coagulation and a biomarker of different degrees of inflammation [9]. It is reported that the level of plasma fibrinogen in patients with ACS was higher than that in healthy controls, and the higher level of plasma fibrinogen might be an independent predictor of major adverse cardiac events during short-term and long-term follow-up [10, 11]. In addition, albumin is inversely related to the degree of inflammatory response [12], and it is an important inhibitor of platelet activation and aggregation [13]. Several evidence indicate that low albumin level is associated with atherosclerotic cardiovascular diseases and related to the major adverse cardiovascular events (MACE) [14, 15]. Recent researches revealed that FAR is efficient in predicting the presence and severity of diseased coronary artery in ST-segment elevation myocardial infarction (STEMI) [16, 17]. In this study, we aim to investigate the relation of FAR with the presence and severity of coronary artery disease in patients with ACS undergoing coronary angiography (CAG).

## Methods

### Study population

From February 2019 to December 2019, a total of 3000 consecutive patients who received electrocardiography (ECG) and CAG due to angina-like chest pain were evaluated and we had access to information that could identify individual participants during or after data collection. ACS was defined based on criteria created by the American College of Cardiology and the European Society of Cardiology and the ACS group was further divided into the unstable angina (UA) group and acute myocardial infarction (AMI) group. As per the inclusion criteria for the control group, patients with normal ECG and without a history of coronary artery disease (CAD) and no stenosis or stenosis degree < 50% on CAG were included. The exclusion criteria of this study included patients with infectious or systemic inflammatory disease, a history of MI or percutaneous coronary intervention (PCI), severe valvular disease, liver and/or renal insufficiency, hematological disorders, thyroid dysfunction and malignant disease. Patients who received the following treatment were also excluded: diuretics, thrombolytic therapy, cytotoxic drugs, corticosteroid and glycoprotein IIb/IIIa inhibitors. Finally, a cohort of 1575 subjects were enrolled in our final analysis. The baseline characteristics and medical history were collected from all subjects.

### Clinical characteristics

Hypertension was defined as office systolic blood pressure (SBP) values ≥ 140 mmHg and/or diastolic blood pressure (DBP) values ≥ 90 mmHg at least two times in different environments [18]. Type 2 diabetes mellitus (T2DM) was defined according to the American Diabetes Association (ADA) criteria: (1) hemoglobin A1c (HbA1c) ≥ 6.5% and/or (2) fasting plasma glucose (FPG) ≥ 126 mg/dL (7.0 mmol/L) and/or (3) 2-h plasma glucose ≥ 200 mg/dL (11.1 mmol/L) during an oral glucose tolerance test (OGTT) and/or (4) in a patient with classic symptoms of hyperglycemia or hyperglycemic crisis, a random plasma glucose ≥ 200 mg/dL [19]. An individual who drank more than 20 g/day was defined as Alcohol drinker. Smokers were defined as those who had smoking regularly over the previous six months.

### Laboratory tests

Venous blood samples were obtained from all participants. The blood parameters were determined by the clinical laboratory of the First Affiliated Hospital of Xi'an Jiaotong University. Levels of white blood cells (WBC), hemoglobin (Hb), platelets (PLT), neutrophils (NEUT) counts, monocyte (MONO) counts, fibrinogen (FIB) were measured on admission. Levels of total cholesterol (TC), triglyceride (TG), high density lipoprotein cholesterol (HDL-C), low density lipoprotein cholesterol (LDL-C), apolipoprotein A (ApoA), apolipoprotein B (ApoB), apolipoprotein E (ApoE), lipoprotein (a) [Lp(a)], serum creatinine (Scr), glucose, blood urea nitrogen (BUN), uric acid (UA), albumin (ALB) were measured after 12 h of overnight fast. The FAR was respectively computed using the absolute fibrinogen count divided by the absolute albumin count.

### Definition of ACS and Gensini score

CAD was defined as 50% luminal diameter stenosis in at least one major epicardial vessel by diagnostic CAG and the diagnostic criteria for ACS recommended by the European Society of Cardiology [20]. In this study, the severity of CAD was determined by the number of diseased vessels and Gensini score (GS). GS is considering the degree of luminal narrowing and the importance of its location, the specific method is as follows: 1 point for less than 25% obstruction, 2 points for 26–50% obstruction, 4 points for 51–75% obstruction, 8 points for 76–90% obstruction, 16 points for 91–99% obstruction, 32 points for complete occlusion (100%). Then the score is multiplied by the factor which depending on the functional significance of the area supplied by that segment. 5 for the left main coronary artery, 2.5 for the proximal segment of left anterior descending artery or circumflex artery, 1.5 for the middle segment of left anterior descending artery, 1 for the apical segment of left anterior descending artery or the middle or distal segment of circumflex artery or the entire segment of the right coronary artery, 0.5 for other small branches of the coronary artery [21]. The result of coronary angiography was reported by two experienced interventional cardiologists. If the viewpoints of the two cardiologists were inconsistent, a third expert was consulted. Another expert interventional cardiologist calculated the scores in all angiograms. Firstly, all patients enrolled were divided into ACS group and control group. Moreover, the patients with ACS were divided into MI group and non-MI group according to their medical history. Finally, the patients with ACS were further classified into the following subgroups including multiple-vessel disease group, high GS group and their matched subgroups in order to evaluate the relation between FAR levels and severity of diseased coronary artery.

### Statistical analyses

The continuous variables were expressed as the mean ± SD, data was compared with the Student’s t-test or Mann–Whitney U test. One-way analysis of variance or Kruskal–Wallis test were performed more than two groups. The category variables were expressed as percentage (%), data was compared with the Chi-square test. FAR was examined in quartiles. Univariate and multivariate logistic regression were used to assess the relationship between different FAR levels and coronary artery severity in patients with ACS. *P* < 0.05 was considered statistically significant. All data were statistically analyzed using the SPSS software version 22.0 for Windows.

## Results

### Baseline characteristics

**Fig. 1:**
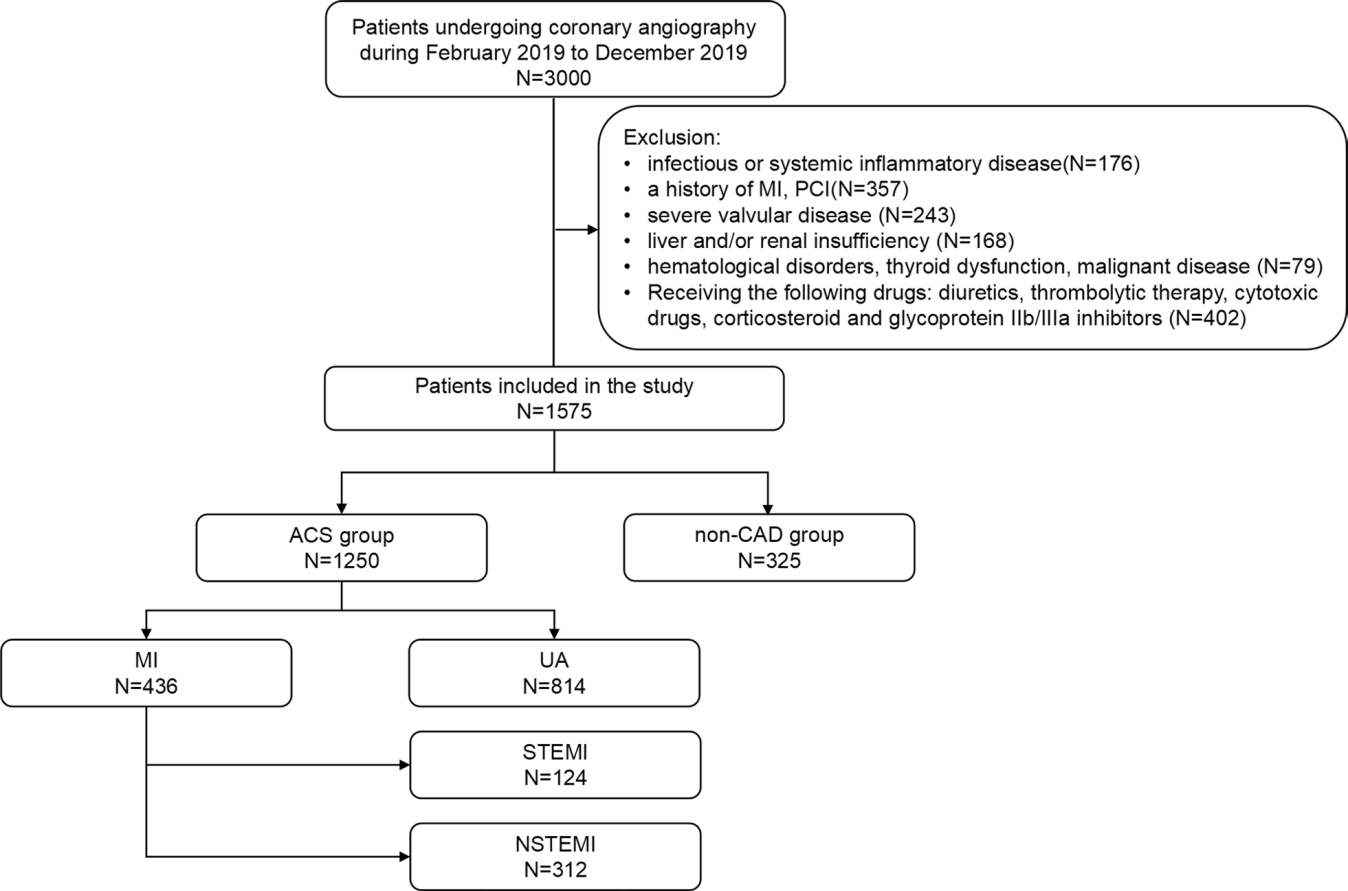
A flow chart of the inclusion and exclusion of patients

A total of 1575 individuals were included in our final analysis according to our inclusion and exclusion criteria (Fig. 1). The individuals were divided into ACS group (n = 1250) and control group (n = 325), the baseline characteristics of the individuals were shown in Table 1. Higher levels of FAR were found in ACS group than that in the non-CAD group (81.20 ± 35.45 vs 72.89 ± 20.24, *P* = 0.001). Moreover, age, levels of the WBC, NEUT, MONO, GLU, ApoA, FIB, prevalence of diabetes mellitus, hypertension, and current smoking were significantly higher in the patients with ACS (*P* < 0.001). Patients with ACS had higher levels of Scr (*P* = 0.001), TG (*P* = 0.049), and Lp(a) (*P* = 0.017), but lower level of ALB (*P* = 0.001) and HDL-C (*P* < 0.001) than patients without CAD. Moreover, the left ventricular ejection fraction (LVEF) was much lower in patients with ACS (*P* < 0.001). There were no significant differences in ALB, TC, ApoB, ApoE between ACS group and the non-CAD group (*P* > 0.05).

**Table 1:** Baseline characteristics of all patients with or without CAD

Characteristics	ACS group (n = 1250)	Non-CAD group (n = 325)	*P* value
Age (years)	60.90 ± 10.11	58.41 ± 9.82	< 0.001
Gender, Male, n (%)	861 (68.8)	154(47.4)	< 0.001
Hypertension, n (%)	727 (58.2)	155(47.7)	0.001
Diabetes, n (%)	326(26.8)	50 (15.4)	< 0.001
Smoker, n (%)	613 (49.0)	104 (32.0)	< 0.001
Alcohol drinker, n (%)	172(13.8)	36 (11.1)	0.203
*Laboratory parameters*			
WBC (× 10^9^/L)	7.38 ± 3.23	5.89 ± 1.51	< 0.001
Hb (g/L)	146.96 ± 15.46	138.93 ± 15.01	0.070
PLT (× 10^9^/L)	206.10 ± 58.51	211.70 ± 57.32	0.388
NEUT (× 10^9^/L)	5.24 ± 2.76	3.89 ± 1.32	< 0.001
MONO (× 10^9^/L)	0.38 ± 0.18	0.30 ± 0.12	< 0.001
FIB (g/L)	3.26 ± 1.07	3.01 ± 0.68	< 0.001
Glucose (mmol/L)	7.56 ± 3.32	6.52 ± 2.54	< 0.001
BUN (mmol/L)	5.71 ± 2.76	5.51 ± 1.46	0.076
Scr (umol/L)	64.85 ± 29.61	60.84 ± 15.15	0.001
UA (umol/L)	332.74 ± 150.30	326.60 ± 146.00	0.510
ALB (g/L)	41.07 ± 4.42	41.97 ± 4.74	0.001
TC (mmol/L)	3.85 ± 0.96	3.83 ± 1.01	0.762
TG (mmol/L)	1.61 ± 1.12	1.47 ± 1.041	0.049
HDL-C (mmol/L)	0.97 ± 0.22	1.05 ± 0.27	< 0.001
LDL-C (mmol/L)	2.28 ± 0.83	2.19 ± 0.74	0.069
APOA (g/L)	1.14 ± 0.20	1.21 ± 0.28	< 0.001
APOB (g/L)	0.81 ± 0.44	0.74 ± 0.20	0.157
APOE (mg/L)	37.36 ± 17.83	37.09 ± 15.12	0.806
Lp(a) (mg/L)	250.93 ± 146.48	216.63 ± 123.25	0.017
FAR	81.20 ± 35.45	72.89 ± 20.24	< 0.001
LVEF (%)	62.3 ± 13.6	66.6 ± 8.8	< 0.001

Values are expressed as mean ± SD or n (%)

SD, Standard deviationl; WBC, white blood cell; Hb, hemoglobin; PLT, platelet; NEUT, neutrophil; MONO, monocyte; FIB, fibrinogen; BUN, urea nitrogen; Scr, serum creatinine; UA, uric acid; TP, total protein; ALB, albumin; TC, total cholesterol; TG, triglyceride; HDL-C, high-density lipoprotein cholesterol; LDL-C, low-density lipoprotein cholesterol; ApoA, apolipoprotein A; ApoB, apolipoprotein B; ApoE, apolipoprotein E; Lp(a), lipoprotein (a); FAR, fibrinogen-to-albumin ratio; LVEF, left ventricular ejection fraction

### Relation of FAR levels and the presence and severity of diseased coronary artery

According to each individual’s CAG, medical history and laboratory examination, the patients with ACS were divided into the following subgroups including MI group, multiple-vessel disease group, high GS group and their matched subgroups in order to further evaluate the relation between FAR levels and severity of diseased coronary artery.

In detail, the patients with ACS were firstly divided into MI group (n = 436) and UA group(n = 814), patients with MI had significantly higher levels of FAR and FIB (FAR 87.97 ± 21.91 vs. 77.57 ± 21.24, *P* < 0.001; FIB 3.44 ± 1.29 g/L vs. 3.16 ± 0.72 g/L, *P* < 0.001), they also had a lower ALB (40.72 ± 5.35 g/L vs. 41.26 ± 3.82 g/L, *P* = 0.038) compared with those in UA group(Table 2). In addition, the patients with MI were divided into STEMI group and Non-ST segment elevation myocardial infarction (NSTEMI) group, patients with STEMI had higher risk of cardiac arrest and lethal arrhythmia (*P* < 0.001), accompanying higher FAR and lower LVEF (FAR 96.77 ± 21.87 vs. 84.48 ± 21.02, *P* = 0.026; LVEF 53.36 ± 9.84 vs. 58.22 ± 10.83, *P* < 0.001) (Additional file 1: Table 1). Then the patients diagnosed with ACS were classified into single-vessel (n = 388), two-vessel (n = 325), and multiple-vessel (≥ 3 vessels, n = 513) group based on CAG. We found that the levels of FAR and FIB in three-vessel group were significantly higher (FAR 85.04 ± 31.53 vs. 83.22 ± 50.93 vs. 74.65 ± 21.98, *P* < 0.001; FIB 3.41 ± 1.03 g/L vs. 3.25 ± 1.02 g/L vs. 3.07 ± 0.81 g/L, *P* < 0.001), and the levels of ALB were lower (ALB 41.06 ± 4.44 g/L vs. 39.72 ± 4.44 g/L vs. 37.46 ± 3.82 g/L, *P* = 0.032) than that in two-vessel and single-vessel group (Table 3). Based on the quartiles of the GS, the patients with ACS were then divided into three groups: low GS (GS < 24, n = 401), intermediate GS (GS 24–58, n = 433) and high GS (GS > 58, n = 416) group (Table 3). In high GS group, the levels of FAR and FIB were significantly higher (FAR 86.10 ± 34.06 vs. 82.65 ± 45.51 vs. 74.55 ± 20.32, *P* < 0.001; FIB 3.45 ± 1.07 g/L vs. 3.26 ± 1.02 g/L vs. 3.06 ± 0.74 g/L, *P* < 0.001), and ALB were lower (ALB 41.17 ± 5.125 g/L vs. 39.67 ± 4.32 g/L vs. 37.41 ± 3.66 g/L, *P* = 0.048) than those in intermediate and low GS groups.

**Table 2:** Baseline characteristics of all patients enrolled with or without MI

Characteristics	MI group (n = 436)	UA group (n = 814)	*P* value
Age (years)	59.18 ± 11.33	61.83 ± 9.28	< 0.001
Gender, Male, n (%)	347(79.6)	514(63.1)	< 0.001
Hypertension, n (%)	227 (52.1)	500 (61.4)	< 0.001
Diabetes, n (%)	91 (20.9)	235 (28.9)	0.001
Smoker, n (%)	265 (60.8)	348 (42.8)	< 0.001
Alcohol drinking, n (%)	59 (13.5)	113 (13.9)	0.931
*Laboratory parameters*			
WBC (× 10^9^/L)	9.51 ± 4.09	6.24 ± 1.82	< 0.001
Hb (g/L)	142.90 ± 17.32	149.31 ± 16.77	0.0494
PLT (× 10^9^/L)	209.47 ± 61.03	204.30 ± 57.07	0.136
NEUT (× 10^9^/L)	7.29 ± 3.30	4.14 ± 1.56	< 0.001
MONO (× 10^9^/L)	0.45 ± 0.22	0.34 ± 0.16	< 0.001
FIB (g/L)	3.44 ± 1.29	3.16 ± 0.72	< 0.001
Glucose (mmol/L)	7.75 ± 3.67	7.46 ± 4.62	0.259
BUN (mmol/L)	5.60 ± 3.87	5.77 ± 1.92	0.301
Scr (umol/L)	67.23 ± 42.94	63.57 ± 18.85	0.037
UA (umol/L)	327.88 ± 219.00	335.34 ± 95.00	0.403
ALB (g/L)	40.72 ± 5.35	41.26 ± 3.82	0.038
TC (mmol/L)	4.01 ± 0.91	3.76 ± 0.97	< 0.001
TG (mmol/L)	1.65 ± 1.05	1.59 ± 1.17	0.374
HDL-C (mmol/L)	0.96 ± 0.21	0.97 ± 0.22	0.262
LDL-C ( mmol/L)	2.44 ± 0.79	2.20 ± 0.84	< 0.001
APOA (g/L)	1.11 ± 0.18	1.16 ± 0.21	< 0.001
APOB (g/L)	0.83 ± 0.22	0.80 ± 0.27	0.582
APOE (mg/L)	38.37 ± 13.07	36.81 ± 19.53	0.196
Lp(a) (mg/L)	263.20 ± 128.57	244.37 ± 155.43	0.140
FAR	87.97 ± 21.91	77.57 ± 21.24	< 0.001
LVEF (%)	54.51 ± 10.33	66.17 ± 13.35	< 0.001

Values are expressed as mean ± SD or n (%)

SD, Standard deviation; WBC, white blood cell; Hb, hemoglobin; PLT, platelet; NEUT, neutrophil; MONO, monocyte; FIB, fibrinogen; BUN, urea nitrogen; Scr, serum creatinine; UA, uric acid; ALB, albumin; TC, total cholesterol; TG, triglyceride; HDL-C, high-density lipoprotein cholesterol; LDL-C, low-density lipoprotein cholesterol; ApoA, apolipoprotein A; ApoB, apolipoprotein B; ApoE, apolipoprotein E; Lp(a), lipoprotein (a); FAR, fibrinogen-to-albumin ratio; LVEF, left ventricular ejection fraction

**Table 3:** Characteristics of all patients enrolled with different severity of diseased coronary artery

Variables	1 vessel (n = 388)	2 vessels (n = 325)	≥ 3 vessels (n = 513)	*P* value
WBC (× 10^9^/L)	6.97 ± 2.95	7.33 ± 2.95	7.77 ± 3.57	0.014
FIB (g/L)	3.07 ± 0.81	3.25 ± 1.02	3.41 ± 1.03	< 0.001
Glucose (mmol/L)	7.03 ± 2.48	7.75 ± 3.70	7.86 ± 3.70	0.012
ALB (g/L)	37.46 ± 3.82	39.72 ± 4.44	41.06 ± 4.44	0.032
TC (mmol/L)	3.76 ± 0.95	3.84 ± 0.93	3.92 ± 0.97	0.040
TG (mmol/L)	1.54 ± 1.34	1.57 ± 0.91	1.67 ± 1.06	0.166
HDL-C (mmol/L)	1.01 ± 0.23	0.96 ± 0.22	0.94 ± 0.21	< 0.001
LDL-C (mmol/L)	2.16 ± 0.78	2.29 ± 0.81	2.36 ± 0.86	0.001
APOA (g/L)	1.17 ± 0.21	1.14 ± 0.21	1.12 ± 0.19	< 0.001
APOB (g/L)	0.74 ± 0.22	0.81 ± 0.57	0.81 ± 0.23	0.009
LVEF (%)	64.25 ± 9.57	62.34 ± 10.91	61.17 ± 11.52	0.016
FAR	74.65 ± 21.98	83.22 ± 50.93	85.04 ± 31.53	< 0.001

	Low GS (< 24)	Intermediate GS (24–58)	High GS (> 58)	*P* value
N	401	433	416	
WBC (× 10^9^/L)	6.38 ± 2.39	7.61 ± 3.72	8.11 ± 3.15	< 0.001
FIB (g/L)	3.06 ± 0.74	3.26 ± 1.02	3.45 ± 1.07	< 0.001
Glucose (mmol/L)	7.15 ± 5.50	7.41 ± 3.32	8.11 ± 3.86	0.004
ALB (g/L)	37.41 ± 3.66	39.67 ± 4.32	41.17 ± 5.125	0.048
TC (mmol/L)	3.74 ± 0.94	3.81 ± 0.88	3.99 ± 1.03	0.001
TG (mmol/L)	1.61 ± 1.35	1.60 ± 1.03	1.62 ± 0.98	0.982
HDL-C (mmol/L)	0.99 ± 0.23	0.96 ± 0.22	0.96 ± 0.22	0.049
LDL-C (mmol/L)	2.14 ± 0.78	2.27 ± 0.75	2.43 ± 0.92	< 0.001
LVEF (%)	66.4 ± 6.9	62.6 ± 18.3	58.9 ± 11.5	< 0.001
FAR	74.55 ± 20.32	82.65 ± 45.51	86.10 ± 34.06	< 0.001

Values are expressed as mean ± SD or n (%)

SD, Standard deviation; WBC, white blood cell; FIB, fibrinogen; ALB, albumin; TC, total cholesterol; TG, triglyceride; HDL-C, high-density lipoprotein cholesterol; LDL-C, low-density lipoprotein cholesterol; FAR, fibrinogen-to-albumin ratio; LVEF, left ventricular ejection fraction

In order to further clarify the relationship between FAR and coronary artery severity in patients with ACS, we divided patients with ACS into three groups according to the quartiles of the FAR levels in the study (quartile1: < 68.27; quartile 2: 68.27–85.46; quartile 3: > 85.46). As shown in Table 4, the proportion of myocardial infarction, multiple vessel lesions and Gensini score increased as the FAR values increased (*P* < 0.001), as well as the proportion of MI, cardiac arrest, lethal arrhythmia (*P* < 0.05). Meanwhile, the levels of FIB and Lp(a) in patients with ACS were gradually tending higher as the FAR quartile increased (*P* < 0.001). However, there were decreasing trend of ALB levels and LVEF in patients with ACS as the FAR quartile increased (*P* < 0.05). There were also significant differences of other variables such as age, gender, WBC, PLT, NEUT, MONO, TC, LDL-C and ApoA and ApoE among these groups (*P* < 0.05). There were no significant differences of other variables such as hypertension, Hb, blood glucose, BUN, Scr, TC, TG and HDL-C among these groups (*P* > 0.05).

**Table 4:** Characteristics of ACS patients in different FAR levels

Characteristics	Quartile 1 (< 68.27)	Quartile 2 (68.27–85.46)	Quartile 3 (> 85.46)	*P* value
N	416	416	418	
Age (years)	58.01 ± 10.20	61.97 ± 9.61	62.72 ± 9.91	< 0.001
Gender, Male, n (%)	324 (77.9)	270 (64.9)	267 (63.9)	< 0.001
Hypertension, n (%)	241 (57.9)	237 (56.9)	249(59.6)	0.326
Diabetes, n (%)	85 (20.4)	113 (27.2)	128 (30.6)	0.001
Smoker, n (%)	222 (53.4)	186 (44.7)	205 (49.0)	0.114
Alcohol drinking, n (%)	59 (14.2)	60 (14.4)	53 (12.7)	0.283
MI, n (%)	119 (28.6)	131 (31.5)	186 (44.5)	< 0.001
NSTEMI, n (%)	111 (26.7)	94 (22.6)	107 (25.6)	0.135
Killip class > 1, n (%)	32 (7.7)	35 (8.4)	34 (8.2)	0.086
Cardiac arrest, n (%)	7 (1.7)	10 (2.4)	14 (3.3)	0.037
Lethal arrhythmia, n (%)	5 (1.2)	11 (2.6)	12 (3.3)	0.042
*Laboratory parameters*				
WBC (× 10^9^/L)	7.54 ± 4.20	6.98 ± 2.61	7.62 ± 2.42	0.008
Hb (g/L)	151.13 ± 18.42	141.03 ± 15.82	148.71 ± 17.18	0.612
PLT (× 10^9^/L)	200.82 ± 58.53	204.4 ± 54.11	213.05 ± 62.05	0.008
NEUT (× 10^9^/L)	5.38 ± 3.30	4.87 ± 2.43	5.47 ± 2.43	0.003
MONO (× 10^9^/L)	0.36 ± 0.20	0.36 ± 0.14	0.42 ± 0.20	< 0.001
FIB (g/L)	2.41 ± 0.54	3.17 ± 0.32	4.19 ± 0.90	< 0.001
Glucose (mmol/L)	7.22 ± 2.93	7.54 ± 3.30	7.92 ± 6.01	0.066
BUN (mmol/L)	5.81 ± 3.97	5.70 ± 1.70	5.61 ± 2.05	0.563
Scr (umol/L)	65.53 ± 42.91	62.85 ± 17.80	66.15 ± 21.72	0.232
UA (umol/L)	414.00 ± 333.39	416.00 ± 323.48	418.00 ± 341.3	0.230
ALB (g/L)	42.87 ± 4.00	41.52 ± 3.57	38.85 ± 4.64	< 0.001
TC (mmol/L)	3.85 ± 1.06	3.92 ± 0.89	3.77 ± 0.91	0.065
TG (mmol/L)	1.63 ± 1.32	1.64 ± 1.07	1.55 ± 0.95	0.436
HDL-C (mmol/L)	0.97 ± 0.22	0.99 ± 0.21	0.95 ± 0.22	0.059
LDL-C (mmol/L)	2.27 ± 0.90	2.34 ± 0.77	2.23 ± 0.80	0.122
APOA (g/L)	1.15 ± 0.19	1.16 ± 0.21	1.10 ± 0.19	< 0.001
APOB (g/L)	0.84 ± 0.40	0.79 ± 0.21	0.80 ± 0.51	0.712
APOE (mg/L)	35.57 ± 18.71	38.76 ± 18.98	35.74 ± 15.51	0.048
Lp(a) (mg/L)	210.09 ± 106.25	245.69 ± 171.56	2956.59 ± 149.75	< 0.001
*Number of diseased vessels, n (%)*				
Single	168(40.4)	122 (29.3)	98 (23.4)	< 0.001
Double	105(25.2)	105(25.2)	115 (27.5)	0.689
Triple	134(32.2)	182 (43.8)	197 (47.1)	< 0.001
LVEF (%)	63.29 ± 10.58	60.51 ± 10.72	57.32 ± 13.47	0.036
Gensini Score	42.00 ± 35.71	50.37 ± 39.52	56.60 ± 37.72	< 0.001

Values are expressed as mean ± SD or n (%)

SD, Standard deviation; WBC, white blood cell; Hb, hemoglobin; PLT, platelet; NEUT, neutrophil; MONO, monocyte; FIB, fibrinogen; BUN, urea nitrogen; Scr, serum creatinine; UA, uric acid; ALB, albumin; TC, total cholesterol; TG, triglyceride; HDL-C, high-density lipoprotein cholesterol; LDL-C, low-density lipoprotein cholesterol; ApoA, apolipoprotein A; ApoB, apolipoprotein B; ApoE, apolipoprotein E; Lp(a), lipoprotein (a); LVEF, left ventricular ejection fraction

**Fig. 2:**
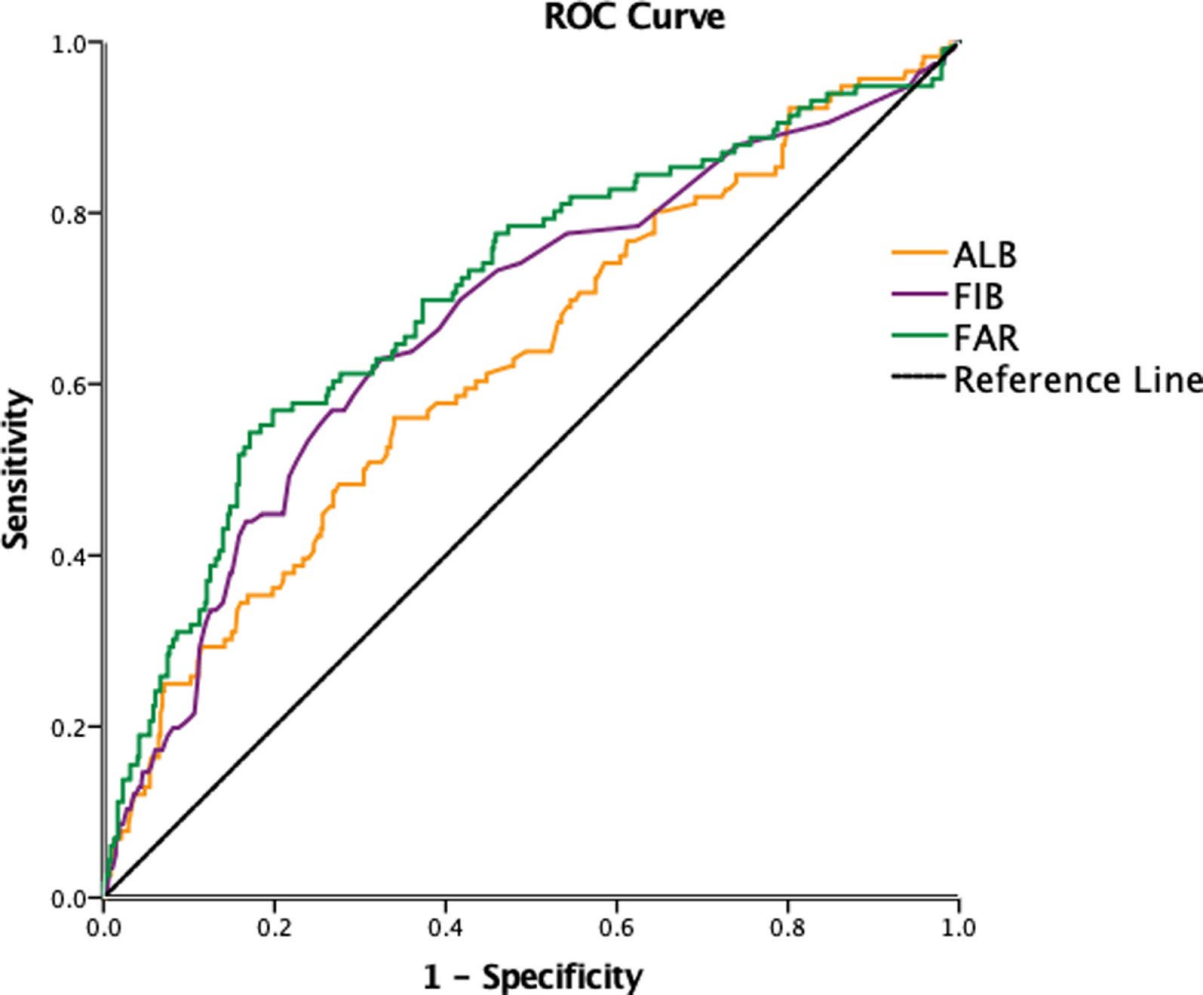
Receiver operating characteristic curve for the diagnostic accuracy of fibrinogen to albumin ratio, fibrinogen and albumin in high Gensini Score

Moreover, in ROC curve analysis FAR had an AUC of 0.706 (95%CI 0.660–0.742) to predict high Gensini sore in patients with ACS, which was statistically significantly better than FIB (AUC 0.663, 95%CI 0.605–0.720, *P* < 0.001) and ALB (AUC 0.617, 95%CI 0.559–0.675, *P* < 0.001) (Fig. 2).

Univariate and multivariate logistic regression analysis of the association between FAR and the severity of coronary artery in patient with ACS are shown in Table 5. In univariate regression analysis, FAR was associated with the severity of diseased coronary artery (MI group vs. non-MI group: OR = 1.744, 95% CI 1.314–2.314, *P* < 0.001; high GS group vs. low GS group: OR = 2.703, 95% CI 1.917–3.810, *P* < 0.001; multiple-vessel disease group vs. single-vessel disease group: OR = 2.520, 95% CI 1.808–3.513, *P* < 0.001). Moreover, even after adjustment for confounders in the multiple regression analysis, such as age, gender, hypertension, diabetes, smoker, alcohol drinking, NEUT, LYM, MONO, glucose, TC, HDL-C, LDL-C and Lp (a), FAR levels remained to be independently associated with the severity of diseased coronary artery (MI group vs. non-MI group: OR = 2.097, 95% CI 1.430–3.076, *P* < 0.001; high GS group vs. low GS group: OR = 2.335, 95% CI 1.567–3.479, *P* < 0.001; multiple-vessel disease group vs. single-vessel disease group: OR = 2.088, 95% CI 1.439–3.030, *P* < 0.001).

**Table 5:** Odd ratios of MI, high GS and multiple-vessel disease in relation to quartiles of FAR

Variables	Quartile 1 (0–68.27)	Quartile 2 (68.27–85.46)	Quartile 3 (> 85.46)
*MI*			
Model 1^a^	1.000 (Ref.)	0.872 (0.648–1.173)	1.744 (1.314–2.314)
*P* value	–	0.364	< 0.001
Model 2^b^	1.000 (Ref.)	1.087(0.796–1.484)	2.298(1.693–3.119)
*P* value	–	0.600	< 0.001
Model 3^c^	1.000 (Ref.)	1.194(0.812–1.758)	2.097 (1.430–3.076)
*P* value	–	0.367	< 0.001
*High GS*			
Model 1^a^	1.000 (Ref.)	1.617 (1.150–2.275)	2.703 (1.917–3.810)
*P* value	–	0.006	< 0.001
Model 2^b^	1.000 (Ref.)	1.739(1.218–2.483)	2.833 (1.981–4.053)
*P* value	–	0.002	< 0.001
Model 3^c^	1.000 (Ref.)	1.637 (1.110–2.414)	2.335 (1.567–3.479)
*P* value	–	0.013	< 0.001
*Multiple-vessel disease*			
Model 1^a^	1.000 (Ref.)	1.870(1.354–2.583)	2.520(1.808–3.513)
*P* value	–	< 0.001	< 0.001
Model 2^b^	1.000 (Ref.)	1.922 (1.373–2.689)	2.516 (1.774–3.569)
*P* value	–	< 0.001	< 0.001
Model 3^c^	1.000 (Ref.)	1.784 (1.256–2.534)	2.088 (1.439–3.030)
*P* value	–	0.001	< 0.001

ORs, Odds ratios; CI, confidence interval

^a^Univariate model

^b^Adjusted for age, gender, hypertension, diabetes, smoker and alcohol drinking

^c^Adjusted for age, gender, hypertension, diabetes, smoker, alcohol drinking, lymphocyte, monocyte, glucose, total cholesterol, high-density lipoprotein cholesterol, low-density lipoprotein cholesterol and lipoprotein (a)

## Discussion

The main finding of our study is that FAR levels are independently associated with CAD and severity of coronary stenosis presented as MI, GS, multiple-vessel disease in patients with ACS. Similarly, patients with more severe coronary stenosis strongly suggest higher FAR levels, an increased level of FAR relatively indicates higher GS in patients with ACS.

Nevertheless, previous studies on the relation of FAR to severity of ACS remain to be further explored. To our best knowledge, no data are currently available regarding between FAR levels and GS, which accepted as a simple, wieldy, and widely used tool in evaluating the severity of coronary stenosis among ACS group. So it is important to understand the relationship between FAR and GS stratification so that physicians can be able to instantly quantitatively evaluate clinical severity in ACS patients admitted to emergency department based on FAR levels which acquired from laboratory tests at admission.

Fibrinogen, a plasma protein produced in the liver, plays an important role in inducing endothelial cell disorganization and migration, stimulates smooth muscle proliferation, and enhances the release of endothelial cell-derived growth factors, which contribute to pathogenesis of vascular inflammation and atherosclerosis [9]. Furthermore, fibrinogen is important for incorporating platelets into a developing thrombus [22]. In addition to the important role of plasma fibrinogen in indicating thrombotic status, recent studies had shown that plasma fibrinogen levels are closely related to the degree of coronary atherosclerosis in patients with stable CAD or STEMI [11, 17]. Tabakc et al. showed that fibrinogen was an easily detected systemic inflammatory marker and was independently associated with coronary severity in patients with CAD [9]. They had demonstrated that plasma fibrinogen with an optimal cut-off value of 411 mg/dL predicts high Syntax score with a sensitivity of 75% and a specificity of 64%. Furthermore, S. Kaptoge et al. found that plasma fibrinogen levels could predict MACE in people at medium cardiovascular risk [23]. However, a meta-analysis revealed that current studies barely supported a causal relationship between fibrinogen and cardiovascular disease, especially in unstable CAD [24]. For the first time, in our study population consisting of ACS, the elevated fibrinogen levels reflect more complex coronary atherosclerosis and may provide evidence for risk stratification of ACS patients at first medical contact.

Albumin, the main protein in human extracellular fluid, plays significant physiological functions as an inflammation biomarker and a mediator of platelet-induced CAD. The relationship between serum albumin and inflammation involved in progression of ACS has been reported in many literatures. Plasma albumin inhibits platelet aggregation by increasing the production of PGD2 in peroxides, and it is shown that low albumin caused more high blood viscosity than plasma fibrinogen or triglyceride by increasing red blood cell lysophosphatidyicholine [13, 25]. Furthermore, some studies and meta-analysis demonstrated that low serum albumin was associated with an increased risk of major adverse cardiovascular events and mortality [15, 26]. Previous data have demonstrated that a lower albumin level is closely related to CAD, but there is currently no literature on the specific relationship in patients with ACS. Our study showed that there were significant difference in plasma albumin levels between the high GS group, MI group and multi-vessel disease subgroups, indicated the relationship between ALB and severity of coronary stenosis in patients with ACS.

Although both plasma fibrinogen and albumin are associated with cardiovascular events, previous studies showed limited evidence about those systemic inflammatory biomarkers alone examining this reciprocal relationship to severity of coronary artery in ACS. Therefore, more and more studies have proposed a combining indicator FAR, which can reflect the state of inflammation and demonstrate this relationship more significantly and more powerfully as a simple index. Current literature have pointed out that FAR has better sensitivity and specificity in predicting MACE than fibrinogen and albumin alone [16, 27]. For example, Xiao L et al. performed a prospective study revealed that preoperative FAR was an independent prognostic factor in STEMI patients undergoing PCI and might improve risk stratification in STEMI [27]. Another study suggested that the value of FAR at admission could be used as an independent predictor of spontaneous recanalization of infarct related arteries in STEMI, and it could be used for early evaluation clinically[28]. Moreover, Karahan et al. found that higher FAR levels were significantly related to higher Syntax score of CAD in patients with STEMI [16]. Occurrence and development of ACS and formation of coronary stenosis are involved unstable plaques and secondary thrombosis result from a series of systemic inflammatory responses. Therefore, the effects of FAR as a combining inflammatory biomarker in patients with ACS remain to be further determined.

In our study, we found that FAR were related to the presence of patients with ACS. In addition, we used Gensini score and conducted several subgroups analyses the relationship between FAR and the severity of coronary artery in ACS. Our study demonstrates that FAR levels are significantly associated with the severity of coronary artery in ACS patients, suggesting that this combined biomarker may capable to be better indicative of severity of ACS.

There were several limitations in this study. Firstly, our article is a single-center with a limited number of participants and a cross-sectional study. Secondly, our study choose Gensini score to evaluate the severity of CAD in ACS, which mainly reflects plaque burden neglecting bifurcation, calcification and distortion of coronary artery compared to Syntax score. Thirdly, the mechanism between FAR and coronary artery severity in patients with ACS is unclear, which needs more studies to investigate.

## Conclusion

The levels of FAR are independently associated with the presence and severity of CAD in patient with ACS. Furthermore, FAR, as a more convenient and rapid biological indicator, may provide a new idea for predicting the presence and severity of ACS.

## Supplementary Information

**Additional file 1:** Baseline characteristics between STEMI and NSTEMI.

## Data Availability

The data that support the findings of this study are available from the corresponding author upon reasonable request.
