# Supplementary material for: Association between fibrinogen-to-albumin ratio and the presence and severity of coronary artery disease in patients with acute coronary syndrome
Source: BMC Cardiovasc Disord. 2021 Dec 7;21:588. doi: 10.1186/s12872-021-02400-z (PMC8650388; doi:10.1186/s12872-021-02400-z)
Supplement: Supplementary file 1 — Additional file 1: Baseline characteristics between STEMI and NSTEMI. [file 12872_2021_2400_MOESM1_ESM.docx]

**Supplementary Table 1** Baseline characteristics between STEMI and NSTEMI.

| **Characteristics** | **STEMI group (n=124)** | **NSTEMI group (n=312)** | **P value** |
| --- | --- | --- | --- |
| **Age (years)** | 59.94±11.30 | 58.88±11.34 | 0.378 |
| **Gender, Male, n (%)** | 95(76.6) | 252(80.8) | 0.200 |
| **Hypertension, n (%)** | 73(58.7) | 154(49.4) | 0.046 |
| **Diabetes, n (%)** | 27 (21.8) | 64 (20.5) | 0.432 |
| **Smoker, n (%)** | 72 (58.1) | 193(61.9) | 0.266 |
| **Alcohol drinking, n (%)** | 13 (10.5) | 46 (14.7) | 0.154 |
| **Killip class>1, n (%)** | 36(29.0) | 65(20.8) | <0.001 |
| **Cardiac arrest, n (%)** | 14(11.3) | 19(6.1) | <0.001 |
| **Lethal arrhythmia, n (%)** | 13(10.5) | 15(4.8) | <0.001 |
| **Laboratory parameters** |  |  |  |
| **WBC (×10^9^/L)** | 8.36±2.47 | 9.97±3.499 | <0.001 |
| **Hb (g/L)** | 141.00±17.35 | 143.66±17.28 | 0.148 |
| **PLT (×10^9^/L)** | 210.58±67.81 | 209.03±58.22 | 0.811 |
| **NEUT (×10^9^/L)** | 6.20±2.35 | 7.73±2.52 | <0.001 |
| **MONO (×10^9^/L)** | 0.44±0.17 | 0.45±0.21 | 0.496 |
| **FIB (g/L)** | 3.67±0.91 | 3.34±1.41 | 0.017 |
| **Glucose (mmol/L)** | 7.51±3.11 | 7.84±3.48 | 0.392 |
| **BUN (mmol/L)** | 5.44±1.48 | 5.66±1.47 | 0.593 |
| **Scr (umol/L)** | 65.14±17.09 | 68.06±19.61 | 0.521 |
| **UA (umol/L)** | 349.37±185.79 | 319.34±185.81 | 0.197 |
| **ALB (g/L)** | 40.63±5.51 | 41.95±5.30 | 0.035 |
| **TC (mmol/L)** | 3.91±0.94 | 4.05±0.89 | 0.150 |
| **TG (mmol/L)** | 1.66±0.95 | 1.64±1.09 | 0.835 |
| **HDL-C (mmol/L)** | 0.94±0.22 | 0.97±0.21 | 0.345 |
| **LDL-C (mmol/L)** | 2.36±0.80 | 2.47±0.78 | 0.183 |
| **APOA (g/L)** | 1.10±0.19 | 1.11±0.17 | 0.612 |
| **APOB (g/L)** | 0.81±0.22 | 0.84±0.21 | 0.208 |
| **APOE (mg/L)** | 37.19±14.71 | 38.84±13.80 | 0.270 |
| **Lp(a) (mg/L)** | 281.89±144.31 | 255.83±122.04 | 0.285 |
| **FAR** | 96.77±21.87 | 84.48±21.02 | 0.026 |
| **LVEF (%)** | 53.36±9.84 | 58.22±10.83 | <0.001 |

Values are expressed as mean ± SD or n (%), SD, standard deviation

#### WBC, white blood cell; Hb, hemoglobin; PLT, platelet; NEUT, [neutrophil](file:///D:/%25E8%25BD%25AF%25E4%25BB%25B6/Dict/8.7.0.0/resultui/html/index.html#/javascript:;); MONO, [monocyte](file:///D:/%25E8%25BD%25AF%25E4%25BB%25B6/Dict/8.7.0.0/resultui/html/index.html#/javascript:;); FIB, fibrinogen; BUN, [urea](file:///D:/%25E8%25BD%25AF%25E4%25BB%25B6/Dict/8.7.0.0/resultui/html/index.html#/javascript:;) [nitrogen](file:///D:/%25E8%25BD%25AF%25E4%25BB%25B6/Dict/8.7.0.0/resultui/html/index.html#/javascript:;); Scr, serum [creatinine](file:///D:/%25E8%25BD%25AF%25E4%25BB%25B6/Dict/8.7.0.0/resultui/html/index.html#/javascript:;); UA, uric acid; ALB, albumin; TC, total cholesterol; TG: triglyceride; HDL-C, high-density lipoprotein cholesterol; LDL-C, low-density lipoprotein cholesterol; ApoA, apolipoprotein A; ApoB, apolipoprotein B; ApoE, apolipoprotein E; Lp(a), lipoprotein (a); FAR, fibrinogen-to-albumin ratio; LVEF, left ventricular ejection fraction.
